# Improving a Secondary Use Health Data Warehouse: Proposing a Multi-Level Data Quality Framework

**DOI:** 10.5334/egems.298

**Published:** 2019-08-02

**Authors:** Sandra Henley-Smith, Douglas Boyle, Kathleen Gray

**Affiliations:** 1 University of Melbourne, AU

**Keywords:** data warehouse, data quality, electronic health records

## Abstract

**Background:**

Data quality frameworks within information technology and recently within health care have evolved considerably since their inception. When assessing data quality for secondary uses, an area not yet addressed adequately in these frameworks is the context of the intended use of the data.

**Methods:**

After review of literature to identify relevant research, an existing data quality framework was refined and expanded to encompass the contextual requirements not present.

**Results:**

The result is a two-level framework to address the need to maintain the intrinsic value of the data, as well as the need to indicate whether the data will be able to provide the basis for answers in specific areas of interest or questions.

**Discussion:**

Data quality frameworks have always been one dimensional, requiring the implementers of these frameworks to fit the requirements of the data’s use around how the framework is designed to function. Our work has systematically addressed the shortcomings of existing frameworks, through the application of concepts synthesized from the literature to the naturalistic setting of data quality management in an actual health data warehouse.

**Conclusion:**

Secondary use of health data relies on contextualized data quality management. Our work is innovative in showing how to apply context around data quality characteristics and how to develop a second level data quality framework, so as to ensure that quality and context are maintained and addressed throughout the health data quality assessment process.

## Introduction

A Data Quality (DQ) framework is essential if we want to be able to assess data quality systematically, according to defined characteristics or dimensions [1,2]. In 1996, Wang and Strong proposed a hierarchical DQ framework that considers data quality in four main categories: intrinsic, contextual, representational, and accessibility; and each of these categories has its own dimensions, e.g. accuracy, within the intrinsic category; relevancy, within the contextual category [1].

Research and redefining of DQ frameworks has progressed since 1996, and industry based DQ frameworks have come into focus, but issues with these frameworks persist. One issue is whether a framework has the flexibility to consider how DQ is assessed in the context of various potential primary and secondary uses. Across such contexts of use, definitions of DQ and its categories and subcategories are not always clear or agreed [2]. The multiple facets to the collection, analysis and use of data are seen with varying biases [3], depending upon the position and viewpoint of the data user; and these contextual perspectives affect perceptions of the quality of data.

In the health industry, Electronic Health Records (EHRs) are increasingly being adopted, and the patient data they hold are becoming the foundation for the safety and quality of patient care. In broad terms, the safety and quality of health care provided by individual clinicians and by organizations is highly regulated, with national safety and quality standards applied across all sorts of health services, but this oversight does not extend to scrutinizing the quality of the data that are actually held within an EHR. For example in Australia, the Royal Australian College of General Practitioners (RACGP) sets standards for General Practitioners and for the operation of General Practice Clinics. While RACGP accreditation looks at how complete the patient record within the EHR is, it does not look at any other dimension of the quality of a patient’s data in the EHR [4]. In an example from another perspective, Australian General Practices, hospitals and other health service providers can choose to implement a variety of information systems to meet their patient care needs, business administration needs and required interactions with health funders, and so various EHR systems are available in the market. Although the vendors are expected to meet certain government requirements [5], there is no overarching mechanism for the accreditation, auditing or regulation of these systems after they are implemented in a health service.

As the health industry moves ahead with EHRs, a data-driven health care system is emerging. This relies on greater secondary use of data – that is, the extraction, compilation and analysis of diverse sets of data from many patients’ records, to answer particular clinical or administrative questions, beyond those raised during the immediate care of any individual patient whose data are recorded. Warehousing data from EHRs for secondary use is becoming more common. Thus it is ever more important to apply quality controls throughout the collection and use of EHR data [3].

Data quality issues faced by secondary users of data extracted from EHR systems include the inconsistent use of coding systems such as SNOMED, ICD10 or other terminology; data may be miscoded if those who initially enter the data do not have the time, skills, or knowledge to ensure accuracy, or if they lack training and support within the workplace or from system vendors. Also, across different EHR systems, there is no common or consistent way to enter data, for example, using free text boxes, radio buttons, or pick lists. Moreover these systems do not always have built-in checks, for example to ensure that a reason for visit or a diagnosis is not miscoded as a procedure or some other incorrect data type [6,7,8]. Furthermore, the precise aim of secondary use of patient data can raise other data quality issues, because data quality has both intrinsic and intentional aspects:

*“Fitness for use does not change the underlying intrinsic DQ features of the elements in a data set; it does change the acceptability of measures of DQ based on the intended use. For example, a completeness measure of 70 percent may be acceptable for a variable that is known to be not relevant to an analysis but would be unacceptable in an analysis where the variable was deemed important.”* [2]

Prospective secondary users of warehoused EHR data need to understand what meaning was associated with the variables and attributes that contain the data they need, in the primary use context where the data were entered. As well they need to understand the clinical practice environment where this EHR system was implemented. Without an understanding of these data collection influences on overall data quality, potential biases that could exist within the data cannot be identified or documented; this will have a flow-on effect on the analysis and interpretation of these data in secondary use. Two kinds of considerations for assessing the fitness for secondary use of data extracted from EHRs – system design and system use – are described next.

When evaluating proposed secondary uses of EHR data, it is important to consider the original design of the EHR system’s database naming conventions, structure and associated user interface, and determine the intended primary use of the applications, forms, screens and fields in relation to where they are stored in the database. When secondary use relies upon a data warehouse of data sourced from many different non-standardized EHR systems, the data warehouse custodians are unlikely to be able to influence the ensuing data quality issues at their source [3]. This is why data quality assurance on the data in the warehouse needs to factor in the EHR systems’ original intent; when contextual information about the user interface of the EHR system is not available, it is important to document occasions where data are most likely to be mis-entered or misrepresented.

The way EHR systems are used in practice needs consideration to ensure that secondary data users are aware of data quality limitations resulting from the way patient data are entered and maintained. EHR system users may lack training or confidence, have limited IT skills or knowledge, or experience time and other pressures in the work environment, all of which may combine to reduce data quality even for primary use [3,6]. Again, the data warehouse custodians are unable to influence such data quality issues directly, and so data quality assurance on the data in the warehouse must take into account potential issues with data that arise from the challenges that EHR system users are known to face.

These two examples underscore the point that data quality assurance needs to occur on the EHR data stored within a secondary use data warehouse, so that we can be confident that they are fit for purpose. We need a systematic way to determine both the intrinsic quality of data, within the primary use EHR system, and as well the intentional quality, in relation to each secondary use objective. Accordingly, health informatics researchers are reviewing DQ frameworks developed within the wider IT industry and other business sectors and are expanding upon these to create DQ frameworks that address the multiple uses and challenges of data extracted from health information systems.

### Objective

We set out to explore how a DQ framework applicable to warehoused EHR data for secondary use could implement broad industry standards of good practice, whilst identifying the intrinsic features and analytic implications of particular EHR data. Our objective was to establish a process for characterizing data quality on a project-by-project basis, enabling us to tailor the data quality assessment to the specifics of each intended secondary use.

## Method

We undertook a literature review to identify the latest research on data quality assurance frameworks. An important development of this kind was a systematic review of DQ frameworks from 1996 to 2013, published in 2016 by Kahn et al. They focused on harmonizing data quality assessment terminology, and they augmented their analysis of the literature by workshops and interviews with health industry participants, to produce a comprehensive framework for the secondary use of EHR data [2]. We determined that the framework provided by Kahn et al. was a sound foundation for our research. Independently, this paper was identified as one of the top Clinical Research Informatics papers in 2017 [9] providing external validation for our decision to build on this work.

We then compiled an initial list of specifications and attributes that we wanted to incorporate in a data quality framework to capture the two key contexts that we consider to be important influences on warehoused EHR data’s fitness for secondary use. This list was based on knowledge gained through the literature review, as well as on extensive prior health data warehouse experience among the authors. This list is shown in Appendix A.

Then we re-analyzed the Khan et al. paper and its top-level framework design, checking for compatibility with the list in Appendix A. From this review, we determined that a two-level framework would allow for the incorporation of contexts:

**Level 1:** Original data (context of representation in the data warehouse): This first level provides metrics to assess the data held in a data warehouse (including any transformations) in terms of their intrinsic quality, based upon the source systems’ use of naming conventions. This is derived from Khan et al.

**Level 2:** Uses of the warehoused data (context of secondary use): This second level matches the context of a secondary user’s data request to the data held within the data warehouse, so that data quality can be assessed based upon the constraints and requirements of the area of interest or question reflected in the data request. This level is designed to have its own defined terminology, definitions and characteristics; while there may be some cross-over from Level 1, there will also be separate attributes and characteristics. In some cases, an attribute may have the same DQ attribute name in both levels of the framework, but a different contextual meaning to define how the attribute should be assessed in the light of each specific data request.

Then, we conducted a 14-step iterative testing and refinement process, to develop a DQ framework that could fully represent the specifications and attributes in Appendix A in a two-level framework, and to translate it into a checklist that could be used on a case-by-case basis:

1. Choose a subset of fields and tables in a data set sourced from various EHR systems and designed for secondary use, against which to test the DQ attributes: The tables and fields chosen should aim to reflect the diversity of EHR data held in the data warehouse.
2. Define an initial Level 1 checklist based on the specifications and attributes of Appendix A in conjunction with Khan et al.
3. Test the checklist against the secondary use dataset.
4. Based on issues identified from testing, re-work the Level 1 DQ framework into a new criteria-plus-checklist template.
5. Perform a dry run with one of the same fields used in Step 1 of this process, for the initial testing of the re-worked Level 1 DQ framework.
6. Test on the rest of the Step 1 fields, the re-worked Level 1 DQ framework and checklist template.
7. Have co-authors regularly review and critique the revision of Level 1.
8. Write up the Level 1 DQ terms definitions, including context and reporting, as the confirmed Level 1 DQ framework.
9. Test the confirmed Level 1 DQ framework checklist template in a simulated real-world EHR data warehouse and document all results.
10. Develop the level 2 DQ terms, definitions and examples based upon the Level 1 DQ terms.
11. Review the initial Level 2 DQ framework based on the context checklist in Appendix A and following the intent of Level 2 defined above.
12. Test the checklist against a data extraction request to a simulated real-world EHR data warehouse, typical of requests for data for secondary use.
13. Write up Level 2 DQ terms definitions, including context and reporting, as the confirmed DQ framework.
14. Test the confirmed DQ framework in a simulated real-world data warehouse and document all results available for data from selected EHR systems.

Level 1 testing was undertaken within a test data warehouse modelled on a real-world data warehouse, on data from two General Practice EHR systems from which data are sourced and stored there; both warehouses are managed by the authors’ research group. The source systems, Medical Director^TM^ and Best Practice^TM^, were prioritized because they are the most widely used General Practice EHR systems in Australia, and also because many data warehouses in Australia source data from these systems by various mechanisms. Tables and fields within these systems were selected for testing based on their diversity of function, and included those designed to record the following data: Patient demographics, Reason for visit, Clinical diagnosis, Current prescriptions including medication name, and Perinatal data; each was assessed in the form of a data field from a CSV file extract from the source system. Level 2 testing used a modification of an actual request to extract de-identified primary care data from a warehouse managed by the authors’ research group.

## Results

This project created and tested a two-level framework-plus-checklist, formatted as re-usable templates, for assessing the data quality of warehoused EHR data for secondary use. For each level, first the framework was refined, then the checklist was developed. Key learnings from the testing process, and details of the resulting template, are described for each level.

### Level 1 Data quality framework and checklist

The Level 1 framework and checklist enables the data’s intrinsic value and their data warehouse context to be clearly documented – regardless of the analytic implications of the data.

Appendix B outlines all the enhancements on Kahn et al.’s existing framework that were made to build the Level 1 sections and sub-sections required for our DQ framework. Through testing we found that revision was required, to describe the DQ framework characteristics that would address the problems the authors had experienced in working with a secondary use data warehouse. For example, we found that within the contextual components of the DQ framework, the context of the data warehouse itself was missing; and some DQ characteristics from Kahn et al. needed modification or removal. Testing and re-working the DQ framework sections and sub-sections resulted in the consolidated requirements shown in Table 1 below.

**Table 1:** Level 1 Data Quality Sections and Sub Sections to be addressed in a DQ framework.

DQ Section and Subsection areas	Explanation

**1. Source System Name**	This is the name of the application where the secondary use data were extracted from and were being assessed for data quality
**2. Data Warehouse Context**	This provides context on the data warehouse environment where the secondary use data are held in relation to the following: Number of source systems Data processing type: Raw data or processed data Data extraction and storage type: Extract all data and over-write what is stored each time, or extract complete data first and then only changes after that Source data extraction type: Database, delimited text file, Excel file, other file type
**3. Table Name 1**	This is the name of the table being assessed for data quality, repeated for each table that is extracted from the source system. The following need to be addressed within the framework: Location of the table context/meaning Location of table fields/variables list Section to list out DQ characteristic assessment requirement Expected result of the characteristic Actual result of the characteristic Result of the characteristic – Pass/Fail If a fail, why did it fail
**4. Field Name 1**	This is the name of the field being assessed for data quality, within the table documented within the table section, repeated for each field within each table that is extracted from the source system. The following need to be addressed within the framework: Location of the field context/meaning Field variable type and length Field input type i.e. look up, text, date/time, integer/numeric Field allowable characters – if other than a look up field Field available variables – if a look up table: Document the variables and associated meanings Location of the look up table variables and meanings documented Section to list out DQ characteristic assessment requirement Expected result of the characteristic Actual result of the characteristic Result of the characteristic – Pass/Fail If a fail, why did it fail Document the data accuracy of the field held within the data warehouse Data interpretation, integrity and limitations Document any data interpretation issues known through the process or known through experience Document any data issues with the data in the data warehouse Document any known data limitations Repeat 4 for Field Name 2 to x, until all fields within the table have been documented Repeat 3 to 4 for Table 2 to x and Field Name 1 to x, until all tables and fields within the system have been documented

The framework requirements outlined in Table 1 helped to inform and develop the Level 1 DQ framework and checklist that is summed up in Table 2. The DQ characteristics that are italicized in Table 2 are derived from Kahn et al.’s DQ framework. The validation requirements are explained using examples that emerged from testing in our simulated data warehouse. The full Level 1 DQ framework and checklist in template are shown in Appendix C.

**Table 2:** Level 1 DQ framework characteristics and validation requirements.

**1. Data Warehouse Context Location Framework**	

**Requirement Description**	**Explanation/Validation Requirements**

**1.1. Data Warehouse (DW) Location**	This is the location of the data warehouse where data are being hosted
**1.2. Number of Source Systems within DW**	This is the number of applications that are being extracted from to output data housed within this DW
**1.3. Data Processed Type**	This is whether the data that are being assessed are from the raw extraction still in the separate source system tables or whether data have been processed and combined into associated tables and fields between the source systems
**1.4. Data Extraction Storage Type**	This is how the data handling of the extraction is done, either extracting all data each time and overwriting what is stored in the DW, or a complete extract when first requested and then extracting updated records only from each of the required tables in subsequent data extractions
**2. Source Database and Table Name Context Location Framework**	

**Requirement Description**	**Validation Requirements**

**2.1. Source System Name**	This is the name of the source database software that is being assessed
**2.2. Source System Extraction Type**	This is how the data were extracted from the source system, i.e. extracted from a CSV file created by the source provider or extracted from a database through the tables or views
**2.3. CSV/Text and Other Source File Additional Information**	Define where or by whom the CSV/text file was created or what the other source of the data is, e.g. from a pathology laboratory CSV file created by the lab’s IT department based upon defined requirements
**2.4. Table/File Name**	This is the name of the database table or CSV/text file that the data has come from as the source that is being assessed
**2.5. Location of the Table Context/Meaning**	This is either the tables context/meaning written or the location of the file that contains this information
**2.6. Location of Table Fields/Variables List**	This is either the tables’ fields listed or the location of the file that contains this information
**3. Table Name Data Quality Framework: *Conformance: Do Data Values Adhere To Specified Standards And Formats?***	

**DQ Characteristic**	**Validation Requirements**

**3.1. Relational Conformance**	
***3.1.1. Data values conform to relational constraints***	The table within the data warehouse should be structured so that it contains easily identifiable fields/columns that can be used as a foreign key, so data are easily linkable in a usable and meaningful way
***3.1.2. Unique (key) data values are not duplicated***	The table must have a unique record ID that is not repeated without easily identifiable reasons, i.e. record has been updated or deleted; a new record has been created for the same record ID; the old record is expired and the status of the new row is set to updated or deleted
***3.1.3. Table from Source System has a Created Date, Created By, Updated Date, Updated By and Record Status fields***	The table contains gold-standard fields that enable auditors and users of the secondary data to know if the data contained within the record have been updated and by whom; to know if the record was active, inactive or deleted at the time of data extraction
***3.1.4. The Source System Gold-Standard Field Names and Associated Status Variable Codes and Meanings: Created Date, Created By, Updated Date, Updated By and Record Status fields***	This lists the system field names held within the table for the Created Date, Created By, Updated Date, Updated By and Record Status fields, including the variable code and associated meaning of the record status field or the location of where this information is held
***3.1.5. Table from Data Warehouse Required Additional Fields have at least one of the following Field Types: Imported Date, Exported Date and Data Warehouse Import Status Fields***	The data warehouse as a gold standard should have in each table and each record when it was exported as a date/time stamp from the source system; imported as a date/time stamp into the data warehouse and the status of each record – to ensure the latest data, or data required at a set date and time, are used as required
***3.1.6. The Data Warehouse Gold-Standard Field Names and Associated Status Variable Codes and Meanings: Imported Date, Exported Date and Data Warehouse Import Status Fields***	This lists the system field names held within the table for the Imported Date, Exported Date and Data Warehouse Import Status fields, including the variable code and associated meaning of the Data Warehouse Import Status field or the location of where this information is held
**If failed why**	
**4. Table Name Data Quality framework: *Plausibility: Are Data Values Believable?***	

**4.1. Uniqueness Plausibility**	
***4.1.1. Data values that identify a single object are not unnecessarily duplicated***.	Data held within the table are not duplicated values, with the exception of updated records and deleted records for a specific record and patient held within a table – i.e. patient’s postcode has changed from 3001 to 3124: a new record with the same record ID but an updated DW import status code exists within the table
**If failed why**	
**4.2. Temporal Plausibility**	
***4.2.1. Observed or derived values conform to expected temporal properties***.	Data held within the table are stored within correct timeframes and events expected – i.e. a patient’s appointment start date and time is before the end date and time of the same appointment
***4.2.2. Sequences of values that represent state transitions conform to expected properties***.	Data held within the table that display events that are required to have multiple entries, have them in the expected sequence and associated values based upon external and internal standards or regulations – i.e. date of an initial immunization precedes date of a booster immunization
***4.2.3. Measures of data value density against a time oriented denominator are expected based on internal knowledge***.	Data held within the table show expected fluctuations for time-orientated events based upon local and external knowledge – i.e. increase in influenza immunizations during flu season
**If failed why**	
**5. Field Name Context Location Framework**	

**Requirement Description**	**Valuation Requirements**

**5.1. Field Name**	This is the name of the field within the source database software’s database table that is being assessed
**5.2. Location of the Field Context/Meaning**	This is either the field’s context/meaning as written or the location of the file that contains this information
**5.3. Field Variable Type and Length**	This is the type of field and the length of the field – i.e. char 60
**5.4. Field Key Type**	This indicates if the field is a primary key, composite primary key or a foreign key. This can be skipped if it is not identified as a key field
**5.5. Field Input Type i.e. look up, text, date, integer/numeric**	This is the allowable data input that the field will accept
**5.6. Field Allowable Characters – if other than a look up field**	This is the ASCI characters that the field will allow to be entered – i.e. a phone number field will only allow numeric values with no spaces. This can be skipped if it is a look up field
**5.7. Field Available Variables – if a look up**	If the field is a look up table, this will list either the table location and joining field of the look up values, if there are greater than ten options, or it will list the variable value and corresponding description. This can be skipped if it is not a look up field
**6. Field Name Data Quality Framework: *Conformance: Do Data Values Adhere to Specified Standards and Formats?***	

**DQ Characteristic**	**Validation Requirements**

**6.1. Value Conformance**	
***6.1.1. Data values conform to internal formatting constraints***.	Data contained within the field need to conform to the required expected field type requirements for the system and external standards where the system is being used – i.e. postcode for Australia needs an integer value, of four integers
***6.1.2. Data values conform to allowable values or ranges***.	The data held within the field must contain only the expected values or ranges that the field allows, based upon what the system has been designed to use – i.e. sex can only allow one numeric value that is translatable or one alpha value that is translatable
**If failed why**	
**6.2. *Computational Conformance***	
***6.2.1. Computed values conform to computational or programming specifications***.	Data held within the field conform to known calculation requirements and can be validated with manual calculation of formulas – i.e. the body mass index calculated within the system yields the same results as a manual calculation with the same values
**If failed why**	
**7. Field Name Data Quality Framework: *Completeness: Are Data Values Present?***	

**7.1. The absence of data values at a single moment in time agrees with local or common expectations**.	Data held within the field are not missing or null/blank based upon expected local and external standard requirements – i.e. sex is expected always to have a value present; work contact phone number can be null/blank as not everyone has one
**7.2. The absence of data values measured over time agrees with local or common expectations**.	Data held within the field are null/blank until the action required to generate a value has occurred, within the expected time frames of the local and external standard requirements – i.e. medical discharge time is missing for three consecutive days
**If failed why**	
**7.3. Atemporal Plausibility**	
***7.3.1. Data values and distributions agree with an internal measurement or local knowledge***.	The data stored within the field are stored and displayed with expected values that local and external standards would suggest are acceptable – i.e. height and weight values are positive and above 0
***7.3.2. Data values and distributions for independent measurements of the same fact are in agreement***.	The data stored within the field are in agreement with external standards and knowledge – i.e. the weight of an adult cannot be below 10
***7.3.3. Logical constraints between values agree with local or common knowledge (includes “expected” missingness)***.	The data stored within the field display expected results based upon local and external knowledge and known facts and common sense – i.e. a patient identified as male does not have a pregnancy documented
***7.3.4. Values of repeated measurement of the same fact show expected variability***.	The data stored within the field compared to data of a similar or same requirement display acceptable variability between the data – i.e. sitting blood pressure taken is within similar ranges such as 160/85 at Time 1 and 145/80 (rather than 85/160) at Time 2
**If failed why**	
**8. Field Name Data Quality Framework Overall Results**	

**8.1. Overall Pass/Fail of the data**	This determines if the data held within the field are based upon the assessment of the above characteristics – i.e. if the data have passed with good data quality or failed with bad data quality
**8.2. Accuracy of the data held within the field (%)**	The percentage of data held within the field that is accurate based upon local knowledge and standards – i.e. % of patients who have a sex associated to them and with the correct values based upon the context of the system
**8.3. Completeness of the data held within the field (%)**	The percentage of data held within the field, which has a value held within the field based upon local knowledge and standards – i.e. % of patients who have a sex associated to them.
**8.4. Data limitations of the data within the field in the data warehouse**	Document the limitations of the data held within the field based upon the context of the system the data were obtained from
**8.5. Data interpretation issues of the data within the field in the data warehouse**	Document how the data that are held within the field and table can be misinterpreted – i.e. the doctor associated to a patient from an imported patient record does not have that doctor name within the user table of the application the data were exported from
**8.6. Data issues of the data within the field in the data warehouse**	Document any issues the data can have from local and internal knowledge of the applications – i.e. Medical Director™ allows a user to code a fever as a procedure
**Other comments/feedback**	Document any other relevant information.
**9. Table Data Quality Framework Overall Results**	

**9.1. Overall Pass/Fail of the data**	This determines if the data held within the table are based upon the assessment of the above characteristics – i.e. if the data have passed with good data quality or failed with bad data quality
**9.2. Accuracy of the data held within the table (%)**	The percentage of data held within the table that is accurate based upon local knowledge and standards – i.e. % of patients who have a sex associated to them and with the correct values based upon the context of the system
**9.3. Completeness of the data held within the table (%)**	The percentage of data held within the table, that has a value held within the table, that is based upon local knowledge and standards – i.e. % of patients who have a sex associated to them
**9.4. Data limitations of the data within the table in the data warehouse**	Document the limitations of the data held within the table based upon the context of the system the data was obtained from
**9.5. Data interpretation issues of the data within the table in the data warehouse**	Document how the data that are held within the table can be misinterpreted – i.e. the doctor associated to a patient from an imported patient record does not have that doctor name within the user table of the application the data were exported from
**9.6. Data issues of the data within the table in the data warehouse**	Document any issues the data can have from local and internal knowledge of the applications – i.e. Medical Director^TM^ allows a user to code a fever as a procedure
**Other comments/feedback**	Document any other relevant information

### Level 2 Data quality framework and checklist

The Level 2 framework and checklist enable the data’s intentional value to be documented clearly. It builds on Level 1, to allow DQ assessment to be done in relation to the specific area of interest or inquiry that has generated a request to extract data from the data warehouse. Developing the second level after testing the first level ensured that the data’s intrinsic value was documented, prior to making a second pass to re-assess DQ in terms of the actual fitness of specific EHR data for the purpose of the request.

Table 3 illustrates how the generally applicable level 2 DQ framework and checklist captured and encompassed all the required metadata, about any given request for warehoused data for secondary use. A worked example of its use follows, in Table 4.

**Table 3:** Level 2 Data Quality Sections and Sub Sections needing to be addressed.

Level 2 DQ Section and Subsection areas	Explanation

**1. Area of interest/question to be investigated**	What is the area of interest/question to be investigated through the data analysis
**2. Area of interest/question and sub question requirement context**	This will provide context on the area of interest/question and what is required to ensure the correct context is being used when assessing the quality of the data in the data warehouse and what needs to be addressed within the framework: Location of the research client’s documented variable/data item list for the data required to be extracted, that contains in generic non-system specific terms: Category/Area i.e. current prescriptions, past prescriptions, past history, reason for visit, patient demographic information Variable/data item within each category/area Number of source systems and the names of the source systems required to be extracted from Location of the client’s documented restrictions on the data to be provided – i.e. age-specific conditions only Data extraction and storage type: extract all data and over-write what is stored each time, or extract complete data first and then only changes after that Source data extraction type: database, delimited text file, Excel file, other file type
**3. Table requirements assessment**	This lists out the tables required to answer the area of interest/question and what needs to be addressed within the framework: Location of the documented mapping between the secondary user’s area/category list and the tables that can be supplied Number of tables that can be supplied for required category/areas Location of the tables that cannot be supplied and justification as to why this is not possible – e.g. not available within the Best Practice™ source system Section to list out DQ characteristic assessment requirements Expected result of the characteristic Actual result of the characteristic Result of the characteristic – Pass/Fail If a fail, why did it fail
**4. Field name 1**	This lists out the tables required to answer the area of interest/question and what needs to be addressed within the framework: Location of the documented mapping between the secondary user’s variable/data item list and the fields that can be supplied Number of fields that can be supplied for required category/areas Location of the documentation containing which fields that cannot be supplied and justification as to why this is not possible – e.g. not available within Best Practice™ Section to list out DQ characteristic assessment requirements Expected result of the characteristic Actual result of the characteristic Result of the characteristic – Pass/Fail If a fail, why did it fail Document the data accuracy of the field held within the data warehouse relating to the secondary user’s area of interest or question only Data interpretation, integrity and limitations Document any data interpretation issues known through the process or known through experience Document any data issues with the data in the data warehouse Document any known data limitations

**Table 4:** Worked example of Level 2 DQ framework characteristics and validation requirements.

**1. Research Question Context Framework**	

**Requirement Description**	**Explanation/Validation Requirements**

**1.1. Brief description of the research area of interest/question**	This is the question to be answered, for which data are needed – i.e. are children prescribed antibiotics when seeing the doctor for a cold?
**1.2. Location of the required areas and associated data variables to be extracted**	This is the location of the document that contains the list of the areas/categories and their associated data variables that are required for the data analysis to help answer the questions – i.e. area/category could be patient demographics, visit reason and their associated data variables could be sex, year of birth, age, reason, date of visit
**1.3. Number and names of source systems data is required from**	This is the name of the source systems and the number required to have data extracted from them – i.e. two General Practice patient management systems (Best Practice™ and Medical Director™ in this instance)
**1.4. Location of any required restrictions to be placed upon the data to be extracted**	This is the location of the document that contains any restrictions based upon human research ethics review board approval of what data may be obtained – i.e. only data between the dates of 01 Jan 2009 and 01 Dec 2016 and patients who are between 0 and 18 years old
**2. Source System Table Assessment Framework**	

**Requirement Description**	**Explanation/Validation Requirements**

**2.1. Location of the mapped areas/categories to be extracted**	This is the location of the document that contains the associated table names to the area/category required from the source system to be extracted – i.e. patient demographics would map to the patient table
**2.2. Location of the areas/categories that are unable to be supplied with justification provided**	This is the location of the document that contains the required area/categories that are not available within the source system/s. Even if data are available in one but not the other, this needs to be documented with a reason why – i.e. Best Practice™ and Medical Director™ are unable to provide illicit drug history data as this category is not recorded anywhere within these systems
**3. Source System Table Name Framework**	

**Requirement Description**	**Explanation/Validation Steps**

**3.1. Client generic area/category name**	This is the name of the area that the client has requested to be extracted
**3.2. Source System name**	This is the name of the source system the table is being extracted from – i.e. Best Practice™ or Medical Director™
**3.3. Source System Table name**	This is the source system’s table name as shown in the database
**4. Table Name Data Quality Framework: Coherence: Do Data Values Adhere to Specified Requirements and Gold Standards?**	

**DQ Characteristic**	**Explanation/Validation Requirements**

**4.1. Data Coherence**	
***4.1.1. Table from source system has a Created Date, Created By, Updated Date, Updated By and Record Status fields***	The table contains gold-standard fields that enable auditors and users of secondary data to know if the data contained within the record have been updated and by whom, and whether the record was active, inactive or deleted at the time of data extraction
***4.1.2. The source system gold-standard field names and associated status variable codes and meanings: Created Date, Created By, Updated Date, Updated By and Record Status fields***	This lists the system field names held within the table for the Created Date, Created By, Updated Date, Updated By and Record Status fields, including the variable code and associated meaning of the record status field or the location of where this information is held
***4.1.3. Table from data warehouse required additional fields have at least one of the following field types: Imported Date, Exported Date and Data warehouse Import status fields***	The data warehouse as a gold standard should have in each table and each record when they were exported as a date/time stamp from the source system, imported as a date/time stamp into the data warehouse and the status of each record to ensure the latest data, or data required at a set date and time, are used
***4.1.4. The data warehouse gold-standard field names and associated status variable codes and meanings: Imported Date, Exported Date and Data Warehouse Import status fields***	This lists the system field names held within the table for the Imported Date, Exported Date and Data Warehouse Import Status fields, including the variable code and associated meaning of the Data warehouse Import Status field or the location of where this information is held
***4.1.5. The delivered data meet expected constraints or restrictions***	The data held within the table are able to meet the required constraints or restrictions to meet the needs of the area of interest or answer the required question/s – i.e. data held within the table contain the details about variant brand names for antibiotics such as amoxycillin
**5. Table Name Data Quality Framework: *Plausibility: Are Data Values Believable?***	

**DQ Characteristic**	**Explanation/Validation Requirements**

**5.1. Uniqueness Compatibility**	
***5.1.1. Data values delivered that identify a single object are not unnecessarily duplicated***.	Data held within the table are not duplicated values with the exception of updated records and deleted records for a specific record and patient held within a table – i.e. patient’s postcode has changed from 3001 to 3124: a new record with the same record ID but an updated Data Warehouse Import Status code exists within the table
**5.2. Temporal Compatibility**	
***5.2.1. Observed or derived values that are delivered conform to expected temporal properties***.	Data held within the table are stored within correct timeframes and events expected – i.e. a patient’s appointment start date and time are before the end date and time of the same appointment
***5.2.2. Observed or derived values that are delivered fall within expected timeframes***	Data held within the table fall within the expected timeframes that the area of interest/question is requiring of the data
**6. Source System Field Assessment Framework**	

**DQ Characteristic**	**Explanation/Validation Requirements**

**6.1. Location of the mapped data variables associated to the area/category to be extracted**	This is the location of the document that contains the associated field names to data variables required from the source system to be extracted – i.e. reason for visit would map to the ‘VisitReason’ table Reason field
**6.2. Location of the data variables that are unable to be supplied with justification provided**	This is the location of the document that contains the required data variables that are not available within the source system/s. Even if it is available in one but not the other, this needs to be documented with a reason why – e.g. Best Practice™ is unable to provide the data variable Country of Birth
**7. Source System Field Name Framework**	

**Requirement Description**	**Explanation/Validation Steps**

**7.1. Client generic data variable name**	This is the name of the data variable that the client has requested to be extracted
**7.2. Source System name**	This is the name of the source system the field is being extracted from -i.e. Best Practice™ or Medical Director™
**7.3. Source System field name**	This is the source system’s field name as shown in the database
**8. Field Name Data Quality Framework: *Conformance: Do Data Values Adhere to Specified Standards and Formats?***	

**DQ Characteristic**	**Explanation/Validation Requirements**

***8.1. Value Conformance***	

***8.1.1. Data values conform to internal formatting constraints***.	Data contained within the field need to conform to the required expected field type requirements for the system and external standards where the system is being used – i.e. postcode for Australia needs an integer value and must be 4 digits
***8.1.2. Data values conform to allowable values or ranges***.	The data held within the field must contain only the expected values or ranges that the field allows, based upon what the system has been designed to use – i.e. sex can only allow 1 numeric value that is translatable or 1 alpha value that is translatable
**8.2. Computational Conformance**	
***8.2.1. Computed values conform to computational or programming specifications***.	Data held within the field conform to known calculation requirements and can be validated with manual calculated formulas – i.e. the body mass index calculated within the system yields the same result as a manual calculation with the same values
**9. Field Name Data Quality Framework: *Completeness: Are Data Values Present?***	

**9.1. The absence of data values at a single moment in time agrees with local or common expectations**.	Data held within the field are not missing or null/blank based upon expected local and external standard requirements – i.e. sex is expected to have a value present always; work contact telephone number can be null/blank as not everyone has one
**9.2. The absence of data values measured over time agrees with local or common expectations**.	Data held within the field are null/blank until an action that generates the value, within the expected time frames of the local and external standard requirements – i.e. medical discharge time is missing for three consecutive days.
**9.3. Atemporal Compatibility**	
***9.3.1. Data values and distributions agree with an internal measurement or local knowledge***.	The data stored within the field are stored and displayed with expected values that local and external standards would suggest are acceptable – i.e. height and weight values are positive and above 0
***9.3.2. Data values and distributions for independent measurements of the same fact are in agreement***.	The data stored within the field are in agreement with external standards and knowledge – i.e. the weight of an adult cannot be below 10
***9.3.3. Logical constraints between values agree with local or common knowledge (includes “expected” missingness)***.	The data stored within the field display expected results based upon local and external knowledge and known facts and common sense – i.e. a patient identified as male does not have a pregnancy documented
***9.3.4. Values of repeated measurement of the same fact show expected variability***.	The data stored within the field compared to data of a similar or same requirement display acceptable variability between the data – i.e. sitting blood pressure taken is within similar ranges such as 160/85 at Time 1 and 145/80 (rather than 85/160) at Time 2
**10. Field Name Data Quality Framework Overall Results**	

**10.1. Overall Pass/Fail of the data**	This determines if the data held within the field are relevant to the area of interest/question based upon the assessment of the above characteristics; if the data are able to address the required area of interest/question without modification; or if potential modification of the data request, or of the research question, might be required to obtain value out of the data provided
**10.2. Accuracy of the data held within the field (%)**	The percentage of data held within the field that can answer the required question/area of interest is accurate based upon local knowledge and standards – i.e. % of patients who have a sex associated to them and with the correct values based upon the context of the system
**10.3. Completeness of the data held within the field (%)**	The percentage of data held within the field that has a value held within the field that can answer the required question/area of interest is complete, based upon local knowledge and standards – i.e. % of patients who have a sex associated to them
**10.4. Data limitations of the data within the field in the data warehouse**	Document the limitations of the data relating to the question/area of interest held within the field based upon the context of the system the data were obtained from
**10.5. Data interpretation issues of the data within the field in the data warehouse**	Document how the data that are held within the field and table can be misinterpreted relating to the question/area of interest – i.e. the doctor associated to a patient from an imported patient record does not have that doctor name held within the user table of the application the data were exported from
**10.6. Data issues of the data within the field in the data warehouse**	Document any issues the data can have relating to the question/area of interest from local and internal knowledge of the applications – i.e. Medical Director™ allows a user to code a fever as a procedure
**Other comments/feedback**	Document any other relevant information relating to the question/area of interest
**11. Table Data Quality Framework Overall Results**	

**11.1. Overall Pass/Fail of the data**	This determines if the data held within the table that are relevant to the area of interest/question based upon the assessment of the above characteristics, are able to yield the answer to the required area of interest/question without modification; or if potential modification/diversion might be required to obtain value out of the data provided
**11.2. Accuracy of the data held within the table (%)**	The percentage of data held within the table that can answer the required question/area of interest is accurate based upon local knowledge and standards – i.e. % of patients who have a sex associated to them and with the correct values based upon the context of the system
**11.3. Completeness of the data held within the table (%)**	The percentage of data held within the table that has a value that can answer the required question/area of interest is complete, that is based upon local knowledge and standards – i.e. % of patients who have a sex associated to them
**11.4. Data limitations of the data within the table in the data warehouse**	Document the limitations of the data held within the table based upon the context of the system the data was obtained from
**11.5. Data interpretation issues of the data within the table in the data warehouse**	Document how the data held within the table relating to the question/area of interest can be misinterpreted – i.e. the doctor associated to a patient from an imported patient record, does not have that doctor name held within the wser table of the application the data was exported from
**11.6. Data issues of the data within the table in the data warehouse**	Document any issues the data relating to the question/area of interest can have, from local and internal knowledge of the applications – i.e. Medical Director™ allows a user to code a fever as a procedure
**Other comments/feedback**	Document any other relevant information relating to the question/area of interest

Next, we describe the data requirements of one typical research request to the data warehouse, show where they need to be mapped to the source EHR systems’ tables and fields for quality assurance, and explain how the Level 2 DQ framework and checklist perform to systematize this process.

The specific research question we tested was: Define the burden of antimicrobial prescribing to children in primary care attributable to sore throat as a presenting condition. Data had to be extracted from the following areas of individual patient records, from multiple EHR systems, to find patients in the desired age range, with an upper respiratory tract infection or sore throat presenting condition, who were prescribed antibiotics: Visit reason/Diagnosis; Prescriptions issued/printed (sometimes known as past scripts); Patient demographic information.

Table 4 summarizes how the level 2 DQ framework and checklist enabled us to examine the nuances of the data requirements of this request. The DQ characteristics italicized in Table 4 are taken from Kahn et al.’s DQ framework; the validation requirements are explained in relation to this particular example of a request for EHR data for secondary use. The full Level 2 DQ framework and checklist in templated form is in Appendix D.

## Discussion

DQ frameworks like that of Khan et al. have worked to accommodate both the intrinsic and contextual quality of data as it appears in a data warehouse, with consideration of how the data ultimately may be utilized. However, given the variety of differing secondary uses of warehoused EHR data, such frameworks may not necessarily support assessment of data quality fully enough for specific use cases.

Our work aimed to build an approach that we could test in a real-world data warehouse, that would have an enhanced ability to evaluate data quality in the context of the data’s many and diverse intended secondary uses.

Our first round of testing highlighted that some of the characteristics that were developed by Kahn et al. were not able to be tested against extracted data held within a simulated data warehouse. Many aspects of the framework did work and were able to effectively assess the data with regard to the source systems’ meaning and context for the tables and fields. However, other areas failed, completely or partially, because they could not query assumptions that might be made when data for secondary use are stored outside of an EHR source system’s own database structures.

Table 5 outlines the sections from Khan et al.’s DQ framework in column 1. It summarizes how we reworked the details – what was retained and what was changed or added – in our Level 1 DQ framework, in column 2. It explains how our Level 1 decisions flowed through to the Level 2 framework we introduced, in column 3.

**Table 5:** Cross walk of similarities and differences across Kahn et al. and our two framework levels.

Khan et al.’s framework categories	Level 1 Revised Khan et al. DQ framework categories comparison	Level 2 New framework categories comparison

**Conformance: Do Data Values Adhere to Specified Standards and Formats?**	Kept this wording	Changed this from Conformance to Coherence in Level 2, kept most of the wording except changed Formats to Gold Standards
** Value Conformance**	– Kept this wording and the two definitions in this section – Changed this to be a field level data quality requirement, rather than the global DQ requirement as in Khan et al. – Did not change anything else apart from adding validation requirements	– Kept this wording and the two definitions in this section – Changed this to be a field level data quality requirement, rather than the global DQ requirement as in Khan et al. – Did not change anything else apart from adding validation requirements
** Relational Conformance**	– Kept this wording and the two definitions in this section – Changed this to be a table level data quality requirement, rather than the global DQ requirement as in Khan et al. – Removed “c. Changes to the data model or data model versioning.” From the Framework, this is due to the fact that at the table and field levels of the database there is no version control in the same way there is with paper forms. The version numbering is for the front end of the system that accesses the data information and stores the information in the database behind – Added that the source system table should have Created and Updated Dates, which does give version control on the data added to the database or changed in the database from a front end application by a user – Added 4 new definitions to handle the aspects of the extracted source table context that were not captured in Khan et al.	Did not keep this in Level 2
** Computational Conformance**	– Kept this wording and the one definition in this section – Changed this to be a field level data quality requirement, rather than the global DQ requirement in Khan et al. – Did not change anything else apart from adding validation requirements	– Kept this wording and the one definition in this section – Changed this to be a field level data quality requirement, rather than the global DQ requirement in Khan et al. – Did not change anything else apart from adding validation requirements
**Completeness: Are Data Values Present?**	– Kept this wording and the two definitions in this section – Changed this to be a field level data quality requirement, rather than the global DQ requirement in Khan et al.	– Kept this wording and the two definitions in this section – Changed this to be a field level data quality requirement, rather than the global DQ requirement in Khan et al.
**Plausibility: Are Data Values Believable?**	Kept this wording	Kept this wording
** Uniqueness Plausibility**	– Kept this wording and the one definition in this section Though updated the wording slightly on this characteristic from what it was originally – Changed this to be a table level data quality requirement, rather than the global DQ requirement in Khan et al.	– Kept the wording for Uniqueness but changed from Plausibility to Compatibility. Kept the one definition in this section. Though updated the wording slightly on this characteristic from what it was originally – Changed this to be a table level data quality requirement, rather than the global DQ requirement in Khan et al.
** Atemporal Plausibility**	– Kept this wording and the four definitions in this section – Changed this to be a field level data quality requirement, rather than the global DQ requirement in Khan et al.	– Kept the wording for Uniqueness but changed from Plausibility to Compatibility. Kept the one definition in this section Though updated the wording slightly on this characteristic from what it was originally – Changed this to be a field level data quality
** Temporal Plausibility**	– Kept this wording and the three definitions in this section. – Changed this to be a table level data quality requirement, rather than the global DQ requirement in Khan et al. – Changed this to be a table level data quality requirement, rather than the global DQ requirement in Khan et al.	– Kept the wording for Uniqueness but changed from Plausibility to Compatibility. Kept the one definition in this section. Though updated the wording slightly on this characteristic from what it was originally – Changed this to be a table level data quality requirement, rather than the global DQ requirement in Khan et al.
**New level 1 and level 2 Sections added**		
** Not in Khan et al**.	Data warehouse context characteristics	Research question context framework
	Source system name and table name context characteristics	Source system table assessment framework
	Table name data quality framework characteristics	Source system table name framework characteristics
		Data coherence added to the source table name framework
	Field name context characteristics	Source system field assessment framework characteristics
	Field name data quality framework characteristics	Source system field name framework characteristics
	Field name data quality framework overall results documented	Field name data quality framework overall results documented
	Table data quality framework overall results documented	Table data quality framework overall results documented

### Limitations

Certain areas of Kahn et al.’s DQ framework did not work as intended – in some cases the reason lay in the EHR system design, and in other cases it lay in the researchers not having all of the relevant clinical information or knowledge. As an example, when testing the ‘DrugName’ field held within the Best Practice™ system’s CurrentRx table we encountered these obstacles:

- Two of the original Kahn et al.’s DQ characteristics in Appendix B checklist were unable to be assessed. These were, ‘3.2.3 Changes to the data model or data model versioning’ and ‘4.2 The absence of data values measured over time agrees with local or common expectations’. The reasons the data were unable to be assessed are:
  1. Systems such as Best Practice™ do not store any of the data as version 1, version 2 etc. Instead of using version controls, they have two date/time fields in the majority of their tables. These fields are used to advise when the record was created and updated. Upon initial creation of the record the created and updated date/time fields are the same;
  2. Data extractions are not always able to be done in real-time. As a result of this we are unable to ensure that time critical events that occurred prior to the data extraction, occurred within the required timeframe. Using a data extraction process such as the DELTA processing method, which transmits changes only after an initial extract and utilizing the data extractions tool and data warehouses Status field, we may be able to determine if an update has occurred to the data along with the database’s Created date and time and the Updated date and time for that record overall.
- The DQ Characteristic 7.3.3/9.3.3, ‘Logical constraints between values agree with local or common knowledge (includes “expected” missingness)’, of the DQ framework template in Appendix C/Appendix D, was unable to be assessed in isolation. It requires further knowledge of drug interactions, allergies and the patient’s history to ensure the correct drug has been prescribed to the patient. This characteristic relies upon the individual who assesses the data having all relevant information and knowledge or being able to obtain this from external sources.
- The DQ Characteristic 4.2.3, ‘Measures of data value density against a time-oriented denominator are expected based on internal knowledge’, of the DQ framework template in Appendix C, was unable to be assessed. The individual who assesses the data would need local and external knowledge of drug names being prescribed at set times of the year. This is unable to be tested without medical knowledge as to whether correct medications have been prescribed.
- The original context sections of the DQ framework checklist as shown in Appendix B, were missing the context of the data warehouse characteristics, along with the method used to extract the data from the source system. This was addressed in the reworked framework.

Through testing the enhanced Level 1 DQ original framework and finding the areas that did not work – and assessing why they did not work and what was missing from the context sections we had added to Kahn et al.’s framework – we were able to rework and re-test as shown in the Level 1 reworked DQ framework template in Appendix C.

The main difference between Level 1 and Level 2 is that within Level 2 there is no requirement for computational conformance for the table to be assessed against, but rather the data need to be assessed against data coherence at the table DQ level. This Level 2 DQ framework performed well when tested and modifications were not required within the confines of this research. Only an adjustment to the explanation/validation steps was performed, to ensure that clear and concise understanding and meaning would be delivered to the user of the framework.

In summary, leveraging the framework developed by Kahn et al. allowed our research to focus on how to apply context around data quality characteristics and how to develop a two level DQ framework to ensure that quality and context are maintained throughout the quality assessment processes in a health data warehouse. Testing the Level 1 framework within a simulated environment proved feasible to highlight issues when applying the contextual components to the DQ framework. Developing and testing the Level 2 framework against a typical real-world research question showed that the Level 1 data quality characteristics and the explanation on how to apply them could be maintained in context in the Level 2 assessment.

This work has shown that it is possible to apply context systematically in assessing data quality. Assessment of the data can be performed when clearly documented parameters, variables and restrictions are outlined prior to reviewing the data at a table and field level, either for generic data quality or to answer questions about data that are specific to a research question or area of interest. Documentation of the overall assessment findings at both level 1 and level 2 and any data issues held within the assessed data could be the basis for reports to recipients of the data from a warehouse. Such reports would help them use these data more carefully to meet their needs, with fewer misinterpretations, misunderstandings and methodological issues.

## Conclusion

Health research increasingly depends on data quality assessment that can review and report the fitness for use of data from electronic health records, not only in the context of their source systems but also in the context of their intended secondary uses. Otherwise, data structure biases, data entry issues, ambiguity about what data represent, and many other varying aspects of data, have the potential to affect the value of health research findings. A multi-level data quality framework such as we have presented in this paper is designed to provide a means for data for secondary use to be assessed in terms of the initial purpose of the data, in terms of transformations made in the delivery and representation of data within a data warehouse, and in terms of the subsequent intentions for secondary uses of the stored data.

Our research reinforces more global principles of data quality in relation to health data specifically: “In a database, the data have no actual quality or value […] they only have potential value that is realized only when someone uses the data to do something useful”; “Data quality is related to use and cannot be assessed independently of the user” [10]. Our work is applicable in principle to other health data warehouses besides our test warehouse. With further refinement it may lead to a consistent, contingent and potentially computational approach to determining health data quality in secondary use contexts; and may improve how such health data are reviewed, assessed and valued.

The framework proposed here needs to be validated in real-world settings. The next stage of our research will apply this framework in the primary care data warehouse of a major biomedical research institute. This stage will refine the framework, potentially with new data quality sections and data quality characteristics that perform better across the two different contextual levels, and that identify and document potential bias that comes with the use of secondary data. This stage will also result in the development and documentation of a routine method to store the EHR table and column meanings and context. We expect that this will evolve to become a practical tool that can be used widely in other data warehouses to curate knowledge about the context and meaning of health data for improved secondary use.

## Additional Files

The additional files for this article can be found as follows:

10.5334/egems.298.s1 Appendix A. Initial level 1 DQ framework sections and sub-sections.

10.5334/egems.298.s2 Appendix B. Initial Level 1 Data quality framework and checklist in template.

10.5334/egems.298.s3 Appendix C. Revised Level 1 Data quality framework and checklist in template.

10.5334/egems.298.s4 Appendix D. Level 2 Data quality framework and checklist in template.
