# Supplementary material for: Improving a Secondary Use Health Data Warehouse: Proposing a Multi-Level Data Quality Framework
Source: EGEMS (Wash DC). 2019 Aug 2;7(1):38. doi: 10.5334/egems.298 (PMC6676919; doi:10.5334/egems.298)
Supplement: Appendix A. — Initial level 1 DQ framework sections and sub-sections. [file egems-7-1-298-s1.pdf]

Appendix A. Initial level 1 DQ framework sections and sub-sections

This list shows initial sections and sub-sections that were identified for inclusion in the DQ framework, prior to any testing being conducted.

1. Source System name
2. Table name 1
    a. Location of the table context/meaning
    b. Location of table fields/variables list
3. Field Name 1
    a. Location of the field context/meaning
    b. Field variable type and length
    c. Field input type i.e. look up, text, date, integer/numeric
    d. Field allowable characters – if other than a look up field
    e. Field available variables – if a look up
        i. If a look up table, variables and meanings documented
        ii. Location of the look up table variables and meanings documented
    f. Section to list out DQ characteristic assessment requirement
        i. Expected result of the characteristic
        ii. Actual result of the characteristic
        iii. Result of the characteristic – Pass/Fail
            1. If a fail why did it fail
    g. Document the data accuracy of the field held within the data warehouse
    h. Data interpretation, integrity and limitations
        i. Document any data interpretation issues known through the process or known through experience
        ii. Document any data issues with the data in the data warehouse
        iii. Document any known data limitations
4. Repeat 3 for Field Name 2 to x, until all fields within the table have been documented
5. Repeat 2 to 4 for Table 2 to x and Field Name 1 to x, until all tables and fields within the system have been documented
