# Supplementary material for: Improving a Secondary Use Health Data Warehouse: Proposing a Multi-Level Data Quality Framework
Source: EGEMS (Wash DC). 2019 Aug 2;7(1):38. doi: 10.5334/egems.298 (PMC6676919; doi:10.5334/egems.298)
Supplement: Appendix B. — Initial Level 1 Data quality framework and checklist in template. [file egems-7-1-298-s2.pdf]

Appendix B. Initial Level 1 Data quality framework and checklist in template

This table shows our first pass at creating the Level 1 DQ framework and checklist in templated form. This was based on testing and enhancing Kahn et al.'s 2016 framework, and it implemented context around only those DQ characteristics outlined in Appendix A. The DQ characteristics that are italicized have been taken from Kahn et al. The validation requirements and their explanation have been made applicable for our DQ framework.

**1. GP System and Table Name Context location checklist**				
Requirement Description	Valuation Requirements	Results		
**1.1. GP System Name**	This is the name of the General Practice (GP) software that is being assessed			
**1.2. Table Name**	This is the name of the table within the GP software's database that is being assessed			
**1.3. Location of the table context/meaning**	This is either the tables context/meaning written or the location of the file that contains this information			
**1.4. Location of table fields/variables list**	This is either the tables fields listed or the location of the file that contains this information			
**2. Field Name Context location**				
Requirement Description	Valuation Requirements	Results		
**2.1. Field Name**	This is the name of the field within the GP software's database table that is being assessed			
**2.2. Location of the field context/meaning**	This is either the fields context/meaning written or the location of the file that contains this information			
**2.3. Field variable type and length**	This is the type of field and the length of the field i.e. char 60			
**2.4. Field key type**	This indicates if the field is a Primary Key, Composite Primary Key or a Foreign Key			
**2.5. Field input type i.e. look up, text, date, integer/numeric**	This is the allowable data input that the field will accept			
**2.6. Field allowable characters – if other than a look up field**	This is the ASCII characters that the field will allow to be entered i.e. a phone number field will only allow numeric values with no spaces. This can be skipped if it is a lookup table			
**2.7. Field available variables – if a look up**	If the field is a lookup table, this will list either the table location and joining field of the lookup values, if there are greater than 10 options, or it will list the variable value and corresponding description			
**3. Field Name Data Quality checklist:** ***Conformance: Do Data Values Adhere To Specified Standards And Formats?***				
DQ Characteristic	Validation requirements	Expected Result	Actual Result	Pass/Fail
3.1. Value Conformance				
3.1.1. Data values conform to internal formatting constraints.	Data contained within the field need to conform to the required expected field type requirements for the system and external standards where the system is being used within i.e. Postcode for Australia needs an integer value and no longer than 4 digits			☐ Pass<br>☐ Fail<br>☐ Unable to test<br>☐ N/A
3.1.2. Data values conform to allowable values or ranges.	The data held within the field must only contain the expected values or ranges that the field allows, based upon what the system has been designed to use i.e. Sex can only allow 1 numeric value that is translatable or 1 Alpha vale that is translatable			☐ Pass<br>☐ Fail<br>☐ Unable to test<br>☐ N/A
If failed why				
**3.2. Relational Conformance**				
3.2.1. Data values conform to relational constraints.	The table should be structured that it contains gold standard relational constraints between the data held within this table and data held within another table i.e. foreign key linkages to relating data such as a patient ID from the patient table and look up tables are used appropriately rather than storing the value			☐ Pass<br>☐ Fail<br>☐ Unable to test<br>☐ N/A
3.2.2. Unique (key) data values are not duplicated.	The table must have a unique record ID that increments automatically and sequentially when a new record is added to the system. The forms this can take are: A Primary Key or a Composite Primary Key			☐ Pass<br>☐ Fail<br>☐ Unable to test<br>☐ N/A

3.2.3. Changes to the data model or data model versioning.	Data held within the table for each record must have versioning attached and data are not updated at incorrect times i.e. Version 1 data does not include medical discharge hour.			☐ Pass<br>☐ Fail<br>☐ Unable to test<br>☐ N/A
**If failed why**				
	3.3. Computational Conformance			
3.3.1. Computed values conform to computational or programming specifications.	Data held within the field conform to known calculation requirements and can be validated with manual required calculated formulas i.e. The BMI calculated within the system yields the same results as a manual calculation with the same values			☐ Pass<br>☐ Fail<br>☐ Unable to test<br>☐ N/A
**If failed why**				
	**4. Field Name Data Quality checklist: *Completeness: Are Data Values Present?***			
4.1. The absence of data values at a single moment in time agrees with local or common expectations.	Data held within the field are not missing or NULL/Blank based upon expected local and external standard requirements i.e. Sex is expected to always have a value present, Work number can be NULL/blank as not everyone has a work contact number			☐ Pass<br>☐ Fail<br>☐ Unable to test<br>☐ N/A
4.2. The absence of data values measured over time agrees with local or common expectations.	Data held within the field are NULL/Blank until an event has been actioned for the value to be required within the expected time frames of the local and external standard requirements i.e. Medical discharge time is missing for three consecutive days.			☐ Pass<br>☐ Fail<br>☐ Unable to test<br>☐ N/A
**If failed why**				
	**5. Field Name Data Quality checklist: *Plausibility: Are Data Values Believable?***			
	5.1. Uniqueness Plausibility			
5.1.1. Data values that identify a single object are not duplicated.	Data held within the table are not duplicated with other data held within the table i.e. Each within an organizations chosen application has its own unique record ID associated to it i.e. A single patient only has 1 record and associated record number in the patient table			☐ Pass<br>☐ Fail<br>☐ Unable to test<br>☐ N/A
**If failed why**				
	5.2. Atemporal Plausibility			
5.2.1. Data values and distributions agree with an internal measurement or local knowledge.	The data stored within the field are stored and displayed with expected values that local and external standards would advise are acceptable i.e. Height and Weight values are positive and above 0			☐ Pass<br>☐ Fail<br>☐ Unable to test<br>☐ N/A
5.2.1. Data values and distributions for independent measurements of the same fact are in agreement.	The data stored within the field are in agreement with external standards and knowledge i.e. The weight of an Adult cannot below 10			☐ Pass<br>☐ Fail<br>☐ Unable to test<br>☐ N/A
5.2.2. Logical constraints between values agree with local or common knowledge (includes "expected" missingness).	The data stored within the field display expected results based upon local and external knowledge and known facts and common sense i.e. A patient that identifies as Male does not have a pregnancy documented			☐ Pass<br>☐ Fail<br>☐ Unable to test<br>☐ N/A
5.2.3. Values of repeated measurement of the same fact show expected variability.	The data stored within the field compared to data of a similar or same requirement display acceptable variability between the data i.e. sitting blood pressure taken is within similar ranges such as Time 1 - 190/20 Time 2 - 190/30 rather than 20/190			☐ Pass<br>☐ Fail<br>☐ Unable to test<br>☐ N/A
**If failed why**				
	5.3. Temporal Plausibility			
5.3.1. Observed or derived values conform to expected temporal properties.	Data held within the table are stored within correct timeframes and event expected results i.e. A patients appointment start date and time is before the end date and time of the same appointment			☐ Pass<br>☐ Fail<br>☐ Unable to test<br>☐ N/A
5.3.2. Sequences of values that represent state transitions conform to	Data held within the table display events that are required to have multiple entries have them in the expected sequence and associated values based upon external and internal standards or			☐ Pass<br>☐ Fail<br>☐ Unable to test

expected properties.	regulations i.e. Date of an initial immunization precedes date of a booster immunization.			☐ N/A
5.3.3. Measures of data value density against a time oriented denominator are expected based on internal knowledge.	Data held within the table show expected fluctuations for time-orientated events based upon local and external knowledge i.e. increase of Flu shot immunisations during Flu season			☐ Pass<br>☐ Fail<br>☐ Unable to test<br>☐ N/A
**If failed why**				
**6. Field Name Data Quality Checklist Overall Results**				
**6.1. Overall Pass/Fail of the data**	This determines if the data held within the field, based upon the assessment of the above characteristics, have passed with good data quality or failed with bad data quality			
**6.2. Accuracy of the data held within the field (%)**	The percentage of data held within the field that is accurate based upon local knowledge and standards i.e. Patients have a Sex associated to them and with the correct values based upon the context of the system			
**6.3. Data limitations of the data within the field in the data warehouse**	Document the limitations of the data held within the field based upon the context of the system the data was obtained from			
**6.4. Data interpretation issues of the data within the field in the data warehouse**	Document how the data can be misinterpreted that is held within the field and table i.e. The Doctor associated to a patient from an imported patient record does not have the doctor held within the User table of the application the data were exported from			
**6.5. Data issues of the data within the field in the data warehouse**	Document any issues the data can have from local and internal knowledge of the applications i.e. Medical Director™ will allow a user to code a Fever as a Procedure			
**Other comments/feedback**	Document any other relevant information			
