# Supplementary material for: Improving a Secondary Use Health Data Warehouse: Proposing a Multi-Level Data Quality Framework
Source: EGEMS (Wash DC). 2019 Aug 2;7(1):38. doi: 10.5334/egems.298 (PMC6676919; doi:10.5334/egems.298)
Supplement: Appendix C. — Revised Level 1 Data quality framework and checklist in template. [file egems-7-1-298-s3.pdf]

Appendix C. Revised Level 1 Data quality framework and checklist in template

The DQ characteristics that are italicized have been taken from Kahn et al. The validation requirements and their explanation are written to fit within our DQ framework.

1. Data warehouse Context location checklist		
**Requirement Description**	**Valuation Requirements**	**Results**
**1.1. Data warehouse (DW) location**	This is the location of the data warehouse where it is being hosted	
**1.2. Number of Source systems within DW**	This is the number of applications that are being extracted from and housed within the one DW	
**1.3. Data processed type**	This is if the data being assessed are from the raw extraction still in the separate source system tables or if they have been processed and data combined into associated tables and fields between the source systems	☐ Raw Data<br>☐ Processed Data
**1.4. Data extraction storage type**	This is how the data handling of the extraction is done, either, extracting all data each time and overwriting what is stored in the DW, or a complete extract when first requested and then only extracting out the new, updated records from each of the required tables in subsequent data extractions.	☐ Extract all data overwrite existing data in DW<br>☐ Extract complete data first time, subsequent extractions data changes only
**2. Source Database and Table Name Context location checklist**		
**Requirement Description**	**Valuation Requirements**	**Results**
**2.1. Source System Name**	This is the name of the Source database software that is being assessed	
**2.2. Source System extraction type**	This is how the data were extracted from the source system i.e. extracted from a CSV file created by the source provider or extracted from a database through the tables or views	☐ Database<br>☐ delimited text file e.g. '.CSV' or '.TXT'<br>☐ Excel file<br>☐ Other
**2.3. CSV/Text and Other source file additional**	Define where or by whom the CSV/Text file was created or what the other source of the data is i.e. Pathology Laboratory CSV file created by the	

**information**	lab's IT department based upon defined requirements	
**2.4. Table/File Name**	This is the name of the database table or CSV/Text file that the data have come from as the source that is being assessed	
**2.5. Location of the table context/meaning**	This is either the tables context/meaning written or the location of the file that contains this information	
**2.6. Location of table fields/variables list**	This is either the tables' fields listed or the location of the file that contains this information	

3. Table Name Data Quality checklist: ***Conformance: Do Data Values Adhere To Specified Standards And Formats?***				
**DQ Characteristic**	**Validation requirements**	**Expected Result**	**Actual Result**	**Pass/Fail**
3.1. Relational Conformance				
3.1.1. Data values conform to relational constraints.	The table within the data warehouse should be structured so that it contains easily identifiable fields/columns that can be used as a foreign key, so data are easily linkable in a useable and meaningful way.			☐ Pass<br>☐ Fail<br>☐ Unable to test<br>☐ N/A
3.1.2. Unique (key) data values are not duplicated.	The table must have a unique record ID that is not repeated without easily identifiable reasons i.e. record has been updated or deleted; a new record has been created for the same record ID; with the old record expired and the status of the new row is set to updated or deleted.			☐ Pass<br>☐ Fail<br>☐ Unable to test<br>☐ N/A
**3.1.3. Table from source system has a Created Date, Created By, Updated Date, Updated By and Record Status fields**	The table contains gold standard fields that enable auditors and users of the secondary data to know if the data contained within the record have been updated and by whom; if the record was active; inactive or deleted at the time of the data extraction			☐ Pass<br>☐ Fail<br>☐ Unable to test<br>☐ N/A
**3.1.4. The source system gold-standard field names**	This lists the system field names held within the table for the Created Date, Created By, Updated Date, Updated By and Record status			☐ Pass<br>☐ N/A

**and associated status variable codes and meanings: Created Date, Created By, Updated Date, Updated By and Record Status fields**	fields, including the variable code and associated meaning of the record status field or the location of where this information is held			
**3.1.5. Table from data warehouse required additional fields have at least one of the following field types: Imported Date, Exported Date and Data warehouse Import status fields**	The data warehouse as a gold standard should have in each table and each record when it was exported as a date/time stamp from the source system; imported as a date/time stamp into the data warehouse and the status of each record to ensure the latest data; or data required at a set date and time, is used			☐ Pass<br>☐ Fail<br>☐ Unable to test<br>☐ N/A
**3.1.6. The data warehouse gold-standard field names and associated status variable codes and meanings: Imported Date, Exported Date and Data warehouse Import status fields**	This lists the system field names held within the table for the Imported Date, Exported Date and Data warehouse Import status fields, including the variable code and associated meaning of the Data warehouse Import status field or the location of where this information is held			☐ Pass<br>☐ N/A
**If failed why**				
**4. Table Name Data Quality checklist: *Plausibility: Are Data Values Believable?***				
4.1. Uniqueness Plausibility				
4.1.1. Data values that identify	Data held within the table are not duplicated values, with the exception of updated records			☐ Pass<br>☐ Fail

a single object are not unnecessarily duplicated.	and deleted records for a specific record and patient held within a table i.e. patient's postcode has changed from 3001 to 3124: a new record with the same record ID but an updated DW import status code exists within the table			☐ Unable to test<br>☐ N/A
If failed why				
4.2. Temporal Plausibility				
4.2.1. Observed or derived values conform to expected temporal properties.	Data held within the table are stored within correct timeframes and events expected, i.e. A patients appointment start date and time is before the end date and time of the same appointment			☐ Pass<br>☐ Fail<br>☐ Unable to test<br>☐ N/A
4.2.2. Sequences of values that represent state transitions conform to expected properties.	Data held within the table display events that are required to have multiple entries, have them in the expected sequence and associated values based upon external and internal standards or regulations i.e. Date of an initial immunization precedes date of a booster immunization.			☐ Pass<br>☐ Fail<br>☐ Unable to test<br>☐ N/A
4.2.3. Measures of data value density against a time oriented denominator are expected based on internal knowledge.	Data held within the table show expected fluctuations for time-orientated events based upon local and external knowledge i.e. increase of Flu shot immunizations during Flu season			☐ Pass<br>☐ Fail<br>☐ Unable to test<br>☐ N/A
If failed why				

**5. Field Name Context location**		
**Requirement Description**	**Valuation Requirements**	**Results**
**5.1. Field Name**	This is the name of the field within the Source Database software's database table that is being assessed	
**5.2. Location of the field context/meaning**	This is either the fields context/meaning written or the location of the file that contains this information	

**5.3. Field variable type and length**	This is the type of field and the length of the field i.e. char 60	
**5.4. Field key type**	This indicates if the field is a Primary Key, Composite Primary Key or a Foreign Key	
**5.5. Field input type i.e. look up, text, date, integer/numeric**	This is the allowable data input that the field will accept	
**5.6. Field allowable characters – if other than a look up field**	This is the ASCI characters that the field will allow to be entered i.e. a phone number field will only allow numeric values with no spaces. This can be skipped if it is a lookup table	
**5.7. Field available variables – if a look up**	If the field is a lookup table, this will list either the table location and joining field of the lookup values, if there are greater than 10 options, or it will list the variable value and corresponding description	

6. Field Name Data Quality checklist: ***Conformance: Do Data Values Adhere To Specified Standards And Formats?***

DQ Characteristic	Validation requirements	Expected Result	Actual Result	Pass/Fail
		6.1. Value Conformance		
6.1.1. Data values conform to internal formatting constraints.	Data contained within the field need to conform to the required expected field type requirements for the system and external standards where the system is being used within i.e. Postcode for Australia needs an integer value, no longer or shorter than 4			☐ Pass<br>☐ Fail<br>☐ Unable to test<br>☐ N/A
6.1.2. Data values conform to allowable values or ranges.	The data held within the field must only contain the expected values or ranges that the field allows, based upon what the system has been designed to use i.e. Sex can only allow 1 numeric value that is translatable or 1 Alpha value that is translatable			☐ Pass<br>☐ Fail<br>☐ Unable to test<br>☐ N/A
**If failed why**				
		6.2. Computational Conformance		
6.2.1. Computed values	Data held within the field conform to known calculation requirements and can be validated			☐ Pass<br>☐ Fail

conform to computational or programming specifications.	with manual required calculated formulas i.e. The BMI calculated within the system yields the same results as a manual calculation with the same values			☐ Unable to test<br>☐ N/A
**If failed why**				
**7. Field Name Data Quality checklist: *Completeness: Are Data Values Present?***				
7.1. The absence of data values at a single moment in time agrees with local or common expectations.	Data held within the field are not missing or NULL/Blank based upon expected local and external standard requirements i.e. Sex is expected to always have a value present. Work number can be NULL/blank as not everyone has a work contact number			☐ Pass<br>☐ Fail<br>☐ Unable to test<br>☐ N/A
7.2. The absence of data values measured over time agrees with local or common expectations.	Data held within the field are NULL/Blank until an event has been actioned for the value to be required within the expected time frames of the local and external standard requirements i.e. Medical discharge time is missing for three consecutive days.			☐ Pass<br>☐ Fail<br>☐ Unable to test<br>☐ N/A
**If failed why**				
7.3. Atemporal Plausibility				
7.3.1. Data values and distributions agree with an internal measurement or local knowledge.	The data stored within the field are stored and displayed with expected values that local and external standards would advise are acceptable i.e. Height and Weight values are positive and above 0			☐ Pass<br>☐ Fail<br>☐ Unable to test<br>☐ N/A
7.3.2. Data values and distributions for independent measurements of the same fact are in agreement.	The data stored within the field are in agreement with external standards and knowledge i.e. The weight of an Adult cannot be below 10			☐ Pass<br>☐ Fail<br>☐ Unable to test<br>☐ N/A
7.3.3. Logical constraints between values agree with	The data stored within the field display expected results based upon local and external			☐ Pass<br>☐ Fail

local or common knowledge (includes "expected" missingness).	knowledge and known facts and common sense i.e. A patient that identifies as Male does not have a pregnancy documented			☐ Unable to test<br>☐ N/A
7.3.4. Values of repeated measurement of the same fact show expected variability.	The data stored within the field compared to data of a similar or same requirement display acceptable variability between the data i.e. sitting blood pressure taken is within similar ranges such as Time 1 - 190/20 Time 2 - 190/30 rather than 20/190			☐ Pass<br>☐ Fail<br>☐ Unable to test<br>☐ N/A
**If failed why**				

8. Field Name Data Quality Checklist Overall Results

**8.1. Overall Pass/Fail of the data**	This determines if the data held within the field are based upon the assessment of the above characteristics i.e. if the data have passed with good data quality or failed with bad data quality	☐ Pass<br>☐ Fail<br>**Results justification:**
**8.2. Accuracy of the data held within the field (%)**	The percentage of data held within the field that is accurate based upon local knowledge and standards i.e. Patients have a Sex associated to them and with the correct values based upon the context of the system	
**8.3. Completeness of the data held within the field (%)**	The percentage of data held within the field that has a value held within the field based upon local knowledge and standards i.e. Patients have a Sex associated to them	
**8.4. Data limitations of the data within the field in the data warehouse**	Document the limitations of the data held within the field based upon the context of the system the data was obtained from	
**8.5. Data interpretation issues of the data within the field in the data warehouse**	Document how the data can be misinterpreted that is held within the field and table i.e. The Doctor associated to a patient from an imported patient record, does not have the doctor held within the User table of the application the data were exported from	
**8.6. Data issues of the data**	Document any issues the data can have from	

within the field in the data warehouse	local and internal knowledge of the applications i.e. Medical Director™ will allow a user to code a Fever as a Procedure	
Other comments/feedback	Document any other relevant information	
**9. Table Data Quality Checklist Overall Results**		
**9.1. Overall Pass/Fail of the data**	This determines if the data held within the table are based upon the assessment of the above characteristics i.e. if the data have passed with good data quality or failed with bad data quality	☐ Pass<br>☐ Fail<br>Results justification:
**9.2. Accuracy of the data held within the field (%)**	The percentage of data held within the table that is accurate based upon local knowledge and standards i.e. Patients have a Sex associated to them and with the correct values based upon the context of the system	
**9.3. Completeness of the data held within the field (%)**	The percentage of data held within the table, that has a value held within the table, that is based upon local knowledge and standards i.e. Patients have a Sex associated to them	
**9.4. Data limitations of the data within the field in the data warehouse**	Document the limitations of the data held within the table based upon the context of the system the data was obtained from	
**9.5. Data interpretation issues of the data within the field in the data warehouse**	Document how the data can be misinterpreted that is held within the table i.e. The Doctor associated to a patient from an imported patient record, does not have the doctor held within the User table of the application the data was exported from	
**9.6. Data issues of the data within the table in the data warehouse**	Document any issues the data can have from local and internal knowledge of the applications i.e. Medical Director will allow a user to code a Fever as a Procedure	
Other comments/feedback	Document any other relevant information	

This list itemizes the modifications, additions and removals of areas within this revision:

- Added the Data warehouse (DW) context as Section 1, to document the following:
    - DW location within the organization that is assessing the data;
    - The number of source systems the DW stores;
    - If the data being assessed are the raw extracted data or the processed cleansed data;
    - The data extraction storage method of defining if the data are overwritten each time an extraction is run and imported or if the data are the complete dataset first time, then only extracting and importing the changes to the data;
- Added into section 2. Source Database and Table Name Context location checklist, the requirement of 2.3 CSV/Text and Other source file additional information, to document the data extraction method from each source database (e.g. database connection, CSV / text, Excel);
- Updated Section 3. Table Name Data Quality checklist: Conformance, to ensure the Table is assessed against the required DQ characteristics, as follows:
    - 3.1 Relation Conformance was moved from the Field DQ checklist section to the Table level DQ checklist section
    - Expanded on the requirements of this section to add in the following:
        - 3.1.3. Table from source system has a Created Date, Created By, Updated Date, Updated By and Record Status fields
        - The source system gold standard field names and associated status variable codes and meanings: Created Date, Created By, Updated Date, Updated By and Record Status fields
        - In a table from data warehouse, required additional fields have at least one of the following field types: Imported Date, Exported Date and Data warehouse Import status fields
        - The data warehouse gold standard field names and associated status variable codes and meanings: Imported Date, Exported Date and Data Warehouse Import status fields. These should be contained within all tables within the warehouse and do not need to be assessed each time under the Field checklist section
    - Removed the Section 3.2 Relational Conformance as shown in Appendix B sub section 3.2.3. Changes to the data model or data model versioning.

    - Added Section 4 Table Name Data Quality checklist: Plausibility, were added with their sub sections in their entirety and without modification to the DQ characteristic
- Section 5 through to Section 8 were added with their sub sections in their entirety and without modification to the DQ characteristic
