# Supplementary material for: Improving a Secondary Use Health Data Warehouse: Proposing a Multi-Level Data Quality Framework
Source: EGEMS (Wash DC). 2019 Aug 2;7(1):38. doi: 10.5334/egems.298 (PMC6676919; doi:10.5334/egems.298)
Supplement: Appendix D. — Level 2 Data quality framework and checklist in template. [file egems-7-1-298-s4.pdf]

Appendix D. Level 2 Data quality framework and checklist in template

The DQ characteristics that are italicized have been taken from Kahn et al. The validation requirements and their explanation are modified to fit our DQ framework.

1. Research Question context requirements		
**Requirement Description**	**Explanation/Validation Requirements**	**Results**
**1.1. Brief description of the research area of interest/ question**	This is what the question is, that data are needed to answer, i.e. do children get antibiotics when seeing the doctor for a cold	
**1.2. Location of the required areas and associated data variables to be extracted**	This is the location of the document that contains the list of the areas/categories and their associated data variables that is required for the data analysis to help answer the questions i.e. area/category could be patient demographics, visit reason and their associated data variables could be sex, year of birth, age, reason, date of visit	
**1.3. Number and names of source systems data is required from**	This is the name of the source systems and the number required to have extracted from i.e. 2 General Practice Patient management systems which are Best Practice™ and Medical Director™	
**1.4. Location of any required restrictions to be placed upon the data to be extracted**	This is the location of the document that contains any restrictions based upon the ethics approval on what data are able to be obtained i.e. only data between the dates of 01 Jan 2009 and 01 Dec 2016 and patients who are between 0 and 18 years old	
**2. Source System table assessment requirements**		
**Requirement Description**	**Explanation/Validation Requirements**	**Results**
**2.1. Location of the mapped areas/categories**	This is the location of the document that contains the associated table names to the area/category required from the source	

to be extracted	system to be extracted i.e. patient demographics would map to the patient table	
2.2. Location of the areas/categories that are unable to be supplied with justification provided	This is the location of the document that contains the required area/categories that are not available within the source system/s, even if it is available in one but not the other, it needs to be documented with a reason why i.e. Best Practice™ and Medical Director™ are unable to provide illicit drug history as this is not recorded anywhere within the system	
**3. Source System table name**		
**Requirement Description**	**Explanation/Validation Requirements**	**Results**
3.1. Client generic area/category name	This is the name of the area that the client has requested to be extracted	
3.2. Source System name	This is the name of the source system the table is being extracted from i.e. BP or MD	
3.3. Source System Table name	This is the source system's table name as shown in the database	

**3. Table Name Data Quality checklist: Coherence: Do Data Values Adhere To Specified Requirements and Gold-Standards?**				
**DQ Characteristic**	**Explanation/Valuation Requirements**	**Expected Result**	**Actual Result**	**Pass/Fail**
**4.1. Data Coherence**				
4.1.1. Table from source system has a Created Date, Created By, Updated Date, Updated By and Record Status fields	The table contains gold-standard fields that enable auditors and users of the secondary data to know if the data contained within the record have been updated and by who; whether the record was active, inactive or deleted at the time of the data extraction			☐ Pass<br>☐ Fail<br>☐ Unable to test<br>☐ N/A
4.1.2. The field names of the data warehouse	This lists the system field names held within the table for the Created Date, Created By, Updated Date, Updated By and Record status			☐ Pass<br>☐ Fail<br>☐ Unable to test

**required additional fields for and associated status variable codes and meanings: Created Date, Created By, Updated Date, Updated By and Record Status fields**	fields, including the variable code and associated meaning of the record status field or the location of where this information is held			☐ N/A
**4.1.3. Table from data warehouse required additional fields have at least one of the following field types: Imported Date, Exported Date and Data warehouse Import status fields**	The data warehouse as a gold-standard should have in each table and each record when it was exported as a date/time stamp from the source system, imported as a date/time stamp into the data warehouse and the status of each record to ensure the latest data, or data required at a set date and time, is used			☐ Pass<br>☐ Fail<br>☐ Unable to test<br>☐ N/A
**4.1.4. The field names of the data warehouse required additional fields for and associated status variable codes and meanings: Imported Date, Exported Date and Data warehouse Import status fields**	This lists the system field names held within the table for the Imported Date, Exported Date and Data warehouse Import status fields, including the variable code and associated meaning of the Data warehouse Import status field or the location of where this information is held			☐ Pass<br>☐ Fail<br>☐ Unable to test<br>☐ N/A
**4.1.5. The delivered data meets expected**	The data held within the table are able to meet the required constraints or restrictions to meet the needs of the area of interest or			☐ Pass<br>☐ Fail<br>☐ Unable to test

constraints or restrictions	answer the required question/s i.e. data held within the table contain the information for antibiotics such as amoxycillin			☐ N/A
If failed why				
**5. Table Name Data Quality checklist: Plausibility: Are Data Values Believable?**				
5.1. Uniqueness Compatibility				
5.1.1. Data values delivered that identify a single object are not unnecessarily duplicated.	Data held within the table are not duplicated values with the exception of updated records and deleted records for a specific record and patient held within a table i.e. patient's postcode has changed from 3001 to 3124: a new record with the same record ID but an updated DW import status code exists within the table			☐ Pass<br>☐ Fail<br>☐ Unable to test<br>☐ N/A
If failed why				
5.2. Temporal Compatibility				
5.2.1. Observed or derived values that are delivered conform to expected temporal properties.	Data held within the table are stored within correct timeframes and events expected i.e. A patients appointment start date and time is before the end date and time of the same appointment			☐ Pass<br>☐ Fail<br>☐ Unable to test<br>☐ N/A
5.2.2. Observed or derived values that are delivered fall within expected timeframes	Data held within the table fall within the expected timeframes that the required area of interest/question is requiring the data to answer			☐ Pass<br>☐ Fail<br>☐ Unable to test<br>☐ N/A
If failed why				
**6. Source System field assessment requirements**				
**Requirement Description**	**Explanation/Validation Requirements**	**Results**		
**6.1. Location of the mapped data variables associated to the**	This is the location of the document that contains the associated field names to data variables required from the source system to be extracted i.e. reason for visit would map to the VisitReason table Reason field			

area/category to be extracted		
6.2. Location of the data variables that are unable to be supplied with justification provided	This is the location of the document that contains the required data variables that are not available within the source system/s, even if it is available in one but not the other, it needs to be documented with a reason why e.g. Best Practice™ is unable to provide the data variable Country of Birth	

7. Source System field name		
**Requirement Description**	**Explanation/Validation Requirements**	**Results**
7.1. Client generic data variable name	This is the name of the data variable that the client has requested to be extracted	
7.2. Source System name	This is the name of the source system the field is being extracted from i.e. Best Practice™ or Medical Director™	
7.3. Source System field name	This is the source systems field name as shown in the database	

8. Field Name Data Quality checklist: ***Conformance: Do Data Values Adhere To Specified Standards And Formats?***				
**DQ Characteristic**	**Explanation/Validation Requirements**	**Expected Result**	**Actual Result**	**Pass/Fail**
*8.1. **Value Conformance***				
8.1.1. ***Data values conform to internal formatting constraints.***	Data contained within the field need to conform to the required expected field type requirements for the system and external standards where the system is being used within i.e. Postcode for Australia needs an integer value and no longer or shorter than 4			☐ Pass<br>☐ Fail<br>☐ Unable to test<br>☐ N/A
8.1.2. ***Data values conform to allowable values or ranges.***	The data held within the field must only contain the expected values or ranges that the field allows, based upon what the system has been designed to use i.e. Sex can only allow 1 numeric value that is			☐ Pass<br>☐ Fail<br>☐ Unable to test<br>☐ N/A

	translatable or 1 Alpha value that is translatable			
**If failed why**				
8.3. Computational Conformance				
8.3.1. Computed values conform to computational or programming specifications.	Data held within the field conform to known calculation requirements and can be validated with manual required calculated formulas i.e. The BMI calculated within the system yields the same results as a manual calculation with the same values			☐ Pass<br>☐ Fail<br>☐ Unable to test<br>☐ N/A
**If failed why**				
**9. Field Name Data Quality checklist:** ***Completeness: Are Data Values Present?***				
9.1. The absence of data values at a single moment in time agrees with local or common expectations.	Data held within the field are not missing or NULL/Blank based upon expected local and external standard requirements i.e. Sex is expected to always have a value present; Work number can be NULL/blank as not everyone has a work contact number			☐ Pass<br>☐ Fail<br>☐ Unable to test<br>☐ N/A
9.2. The absence of data values measured over time agrees with local or common expectations.	Data held within the field are NULL/Blank until an event has been actioned for the value to be required within the expected time frames of the local and external standard requirements i.e. Medical discharge time is missing for three consecutive days.			☐ Pass<br>☐ Fail<br>☐ Unable to test<br>☐ N/A
**If failed why**				
**9.3. Atemporal Plausibility**				
9.3.1. Data values and distributions agree with an internal measurement or local knowledge.	The data stored within the field are stored and displayed with expected values that local and external standards would advise are acceptable i.e. Height and Weight values are positive and above 0			☐ Pass<br>☐ Fail<br>☐ Unable to test<br>☐ N/A
9.3.2. Data values and	The data stored within the field are in			☐ Pass

distributions for independent measurements of the same fact are in agreement.	agreement with external standards and knowledge i.e. The weight of an Adult cannot be below 10			☐ Fail<br>☐ Unable to test<br>☐ N/A
9.3.3. Logical constraints between values agree with local or common knowledge (includes "expected" missingness).	The data stored within the field display expected results based upon local and external knowledge and known facts and common sense i.e. A patient that identifies as Male does not have a pregnancy documented			☐ Pass<br>☐ Fail<br>☐ Unable to test<br>☐ N/A
9.3.4. Values of repeated measurement of the same fact show expected variability.	The data stored within the field compared to data of a similar or same requirement display acceptable variability between the data i.e. sitting blood pressure taken is within similar ranges such as Time 1 - 190/20 Time 2 - 190/30 rather than 20/190			☐ Pass<br>☐ Fail<br>☐ Unable to test<br>☐ N/A
**If failed why**				

10. Field Name Data Quality Checklist Overall Results		
**10.1. Overall Pass/Fail of the data**	This determines if the data held within the field that is relevant to the area of interest/question based upon the assessment of the above characteristics, are able to deliver/potentially answer the required area of interest/question without modification; or if potential modification/diversion might be required to obtain value out of the data provided	☐ Pass<br>☐ Fail<br>Results justification:
**10.2. Accuracy of the data held within the field (%)**	The percentage of data held within the field that can answer the required question/area of interest is accurate based upon local knowledge and standards i.e. Patients have a Sex associated to them and with the correct values based upon the context of the system	
**10.3. Completeness of the data held within the field**	The percentage of data held within the field that has a value held within the field that can answer the required question/area of interest is complete,	

(%)	based upon local knowledge and standards i.e. Patients have a Sex associated to them	
**10.4. Data limitations of the data within the field in the data warehouse**	Document the limitations of the data relating to the question/area of interest held within the field based upon the context of the system the data was obtained from	
**10.5. Data interpretation issues of the data within the field in the data warehouse**	Document how the data can be misinterpreted relating to the question/area of interest that is held within the field and table i.e. The Doctor associated to a patient from an imported patient record, does not have the doctor held within the User table of the application the data was exported from	
**10.6. Data issues of the data within the field in the data warehouse**	Document any issues the data can have relating to the question/area of interest from local and internal knowledge of the applications i.e. Medical Director will allow a user to code a Fever as a Procedure	
**Other comments/feedback**	Document any other relevant information relating to the question/area of interest	
**11. Table Data Quality Checklist Overall Results**		
**11.1. Overall Pass/Fail of the data**	This determines if the data held within the table that is relevant to the area of interest/question based upon the assessment of the above characteristics, are able to deliver/potentially answer the required area of interest/question without modification; or if potential modification/diversion might be required to obtain value out of the data provided	☐ Pass<br>☐ Fail<br>Results justification:
**11.2. Accuracy of the data held within the field (%)**	The percentage of data held within the table that can answer the required question/area of interest is accurate based upon local knowledge and standards i.e. Patients have a Sex associated to them and with the correct values based upon the context of the system	
**11.3. Completeness of the data held within the field (%)**	The percentage of data held within the table that has a value that can answer the required question/area of interest is complete, that is based upon local knowledge and standards i.e. Patients	

	have a Sex associated to them	
**11.4. Data limitations of the data within the field in the data warehouse**	Document the limitations of the data held within the table based upon the context of the system the data was obtained from	
**11.5. Data interpretation issues of the data within the field in the data warehouse**	Document how the data relating to the question/area of interest can be misinterpreted that is held within the table i.e. The Doctor associated to a patient from an imported patient record, does not have the doctor held within the User table of the application the data was exported from	
**11.6. Data issues of the data within the table in the data warehouse**	Document any issues the data relating to the question/area of interest can have from local and internal knowledge of the applications i.e. Medical Director™ will allow a user to code a Fever as a Procedure	
**Other comments/feedback**	Document any other relevant information relating to the question/area of interest	
